# Rodent Models of Spondyloarthritis Have Decreased White and Bone Marrow Adipose Tissue Depots

**DOI:** 10.3389/fimmu.2021.665208

**Published:** 2021-06-02

**Authors:** Giulia Furesi, Ingrid Fert, Marie Beaufrère, Luiza M. Araujo, Simon Glatigny, Ulrike Baschant, Malte von Bonin, Lorenz C. Hofbauer, Nicole J. Horwood, Maxime Breban, Martina Rauner

**Affiliations:** ^1^ Department of Medicine III & Center for Healthy Aging, Technische Universität Dresden, Dresden, Germany; ^2^ Laboratoire Infection et inflammation, UMR U1173 INSERM/Université de Versailles-Saint-Quentin-Paris-Saclay, Montigny-le-Bretonneux, France; ^3^ Laboratoire d’Excellence Inflamex, Université Paris Descartes, Paris, France; ^4^ Service de Rhumatologie, Hôpital Ambroise Paré, AP-HP, Boulogne, France; ^5^ Department of Medicine I, Technische Universität Dresden, Dresden, Germany; ^6^ Norwich Medical School, University of East Anglia, Norwich, United Kingdom

**Keywords:** spondyloarthritis, HLA-B27 transgenic rat, SKG mouse, bone marrow fat, IL-17

## Abstract

Bone marrow adipose tissue (BMAT) has recently been recognized as a distinct fat depot with endocrine functions. However, if and how it is regulated by chronic inflammation remains unknown. Here, we investigate the amount of white fat and BMAT in HLA-B27 transgenic rats and curdlan-challenged SKG mice, two well-established models of chronic inflammatory spondyloarthritis (SpA). Subcutaneous and gonadal white adipose tissue and BMAT was reduced by 65-70% and by up to 90% in both experimental models. Consistently, B27 rats had a 2-3-fold decrease in the serum concentrations of the adipocyte-derived cytokines adiponectin and leptin as well as a 2-fold lower concentration of triglycerides. The bone marrow of B27 rats was further characterized by higher numbers of neutrophils, lower numbers of erythroblast precursors, and higher numbers of IL-17 producing CD4^+^ T cells. IL-17 concentration was also increased in the serum of B27 rats. Using a cell culture model, we show that high levels of IL-17 in the serum of B27 rats negatively impacted adipogenesis (-76%), an effect that was reversed in the presence of neutralizing anti-IL-17 antibody. In summary, these findings show BMAT is severely reduced in two experimental models of chronic inflammatory SpA and suggest that IL-17 is involved in this process.

## Introduction

Bone marrow adipose tissue (BMAT) has been recently recognized as a unique fat depot (1, 2). While white adipose tissue is crucial for systemic metabolic homeostasis and brown adipose tissue controls adaptive thermogenesis, the role of BMAT remains incompletely understood. Previous studies have shown that bone marrow adipocytes (BMAds) are morphologically similar to white adipocytes with large unilocular lipid droplets, albeit transcriptional analysis revealed a detectable expression of brown adipocyte markers as well (3, 4). Also, BMAds have been recognized as metabolically active cells that secrete adipokines, growth factors, and proinflammatory cytokines (5, 6). As such, BMAds may participate in the immune response and tissue remodeling. In fact, BMAds have been recently shown to control bone remodeling and bone mass maintenance (7, 8). In adulthood, BMAT can account for up to 70% of the bone marrow cavity, and it is further increased in diverse clinical conditions, including obesity, type 2 diabetes, anorexia, and osteoporosis (9, 10).

Although dysregulation of white adipose tissue is a common feature of chronic inflammatory disorders, the impact of long-term inflammation on BMAT remains elusive. In this study, we used spondyloarthritis (SpA) as prototypical chronic inflammatory disorders to investigate how BMAds are affected by chronic inflammation. SpA is a group of chronic inflammatory rheumatic diseases that affect the axial skeleton, peripheral joints, and entheses, leading to bone erosions followed by bony ankylosis (10). In addition, the spectrum of SpA comprises frequent extra-articular features, including anterior uveitis, psoriasis, and inflammatory bowel disease (IBD) (11). The exact etiology and pathogenesis of SpA are still under investigation. Among various genetic (e.g. HLA-B27 association) and immunological factors that trigger the disease, adaptive immune cells such as CD4^+^ T cells have been recognized as key drivers of chronicity in SpA (12). Of note, numerous studies confirmed the critical role of interleukin-17 (IL-17) and T helper (Th) 17 cell subset in joint inflammation and disruption of bone remodeling in arthritis *via* the modulation of matrix metalloproteinases and stimulation of RANKL (13–15). In particular by attracting neutrophils and macrophages to the site of inflammation and enhancing the production of pro-inflammatory cytokines, IL-17 plays a significant role in the pathogenesis of SpA (13, 16). Recently, antibodies neutralizing IL-17, such as secukinumab and ixekizumab, became clinically available to treat SpA, thereby providing new efficacious therapeutic regimens for these disorders (17).

To assess how chronic inflammation may affect BMAT, we utilized HLA-B27 transgenic rats (B27) and curdlan-challenged SKG mice, two well-established models of SpA (18). Both models recapitulate typical features of human SpA, including chronic systemic inflammation and accelerated bone loss and reduced bone strength due to stimulation of osteoclastogenesis and suppression of bone formation (19–21). Our results show that chronic inflammation in these models is associated with a decreased amount of white adipose tissue and BMAT, with a significant decrease of adipokines, such as leptin and adiponectin. Moreover, we show in a cell culture model that high levels of pro-inflammatory IL-17, which is detected in the serum of B27 rats, may contribute to the suppression of adipogenesis. Thus, chronic inflammation has a significant negative impact on BMAT. Whether the loss of BMAT is due to inhibition of BMAT expansion or depletion of existing stores remains to be investigated.

## Methods

### Animals

Two and six months old non-transgenic (NTG) and disease-prone HLA-B27/ hβ_2_m transgenic rats (33-3 line, Fischer (F344) background) of mixed gender were used in this study. Rat lines were bred and maintained under conventional conditions at University of Versailles-St-Quentin. Rats were kept at 21 degree Celsius with a hygrometry of 41%. The 12 hours light cycle is with light on 8AM to 8 PM and off 8PM to 8AM. Two to three rats were kept in cages. All animals received water and food ad libitum (chow: M25, Special Diet Service, Paris, France). Study procedures were approved by the institutional animal care committee (APAFIS-8910). After sacrifice, rats were weighed. Subcutaneous and peri-gonadal fat mass was excised and weighed. Disease activity score was graded from 0-3: 0=no inflammation, 1=arthritis on one leg, 2=arthritis on both legs, 3=arthritis, and other clinical manifestations (orchitis, alopecia, gut inflammation).

Experiments for the SKG model of AS were conducted under a UK Home Office project license (PBFB4BA22) as previously described (22). Mice were kept on a 12 hour light/12 hour dark cycle at 21°C+/- 2°C with 55% humidity +/- 10%. Up to 5 mice were kept per cage with a mixture of control and curdlan-challenged mice in each cage. All animals received water and food ad libitum (chow: RM3 from Special Diet Service; LBS Biotech, UK). BALB/c ZAP-70^W163C^-mutant (SKG) mice were a kind gift from S. Sakaguchi (University of Kyoto, Kyoto, Japan), bred in house and maintained under specific pathogen-free conditions. Disease was induced in 10-week-old female mice using a single dose of 3 mg curdlan (β-1,3-glucan derived from *Alcaligenes faecalis* variety *myxogenes*; Wako, Japan) administered intraperitoneally (i.p.). Arthritis was scored twice a week using the following defined criteria: 0, no joint swelling; 0.5, mild swelling of wrist or ankle, 1.0, pronounced swelling of wrist or ankle, 1.5 including swelling of at least one digit, 2 including swelling of all digits, 3 severe swelling with ankylosis. All paws for each mouse were scored and were summed per mouse to give an overall clinical grade for each mouse ranging from 0 to 12. Ankle diameter and weight were measured twice weekly. After 3 and 6 weeks, mice were sacrificed for organ collection.

### Hematologic Analysis

Peripheral blood was collected from rats by heart puncture into heparinized capillary tubes, and complete blood counts were performed (Sysmex, Paris, France). Hematopoietic cells in the bone marrow were assessed using a bone marrow smear. For that purpose, bone marrow was flushed from the tibia and transferred to an objective slide. Bone marrow was spread using a coverslip and after staining with May-Grünwald, cells were counted in a blinded fashion by two experienced technicians from the hematology department according to their cytomorphological appearance.

### Histology

Subcutaneous and gonadal fat pads, the liver, and the decalcified femur and fourth lumbar vertebrae were fixed in 4% PBS-buffered paraformaldehyde, dehydrated using an ascending ethanol series, and embedded with paraffin. Sections (4 µm) were prepared and stained for hematoxylin/eosin (HE) to assess tissue structures. Adipocyte area and number of adipocytes were quantified using the Osteomeasure software (Osteometrics, Decatur, USA).

### µCT Analysis of Bone Marrow Adipose Tissue Content

BMAT content was analyzed at the distal femur using osmium staining and subsequent µCT analysis (vivaCT40, Scanco Medical, Brüttisellen, Switzerland). Femurs from mice and rats were fixed with 10% PBS-buffered formalin for 24 h and decalcified in Osteosoft (Merck, Darmstadt, Germany). After decalcification, bones were scanned using the vivaCT40 using a standard protocol to ensure complete demineralization. Afterward, bones were incubated with 2% osmium tetroxide (Sigma-Aldrich, Mannheim, Germany) dissolved in 0.1 M sodium cacodylate buffer (Sigma-Aldrich, Mannheim, Germany) for 1 h at room temperature. Specimens were washed and immediately scanned using the µCT with an X-ray energy of 55 keV, 300 ms integration time, and an isotropic voxel size of 10.5 µm. The fat volume (FV) in the rats was calculated from 700 slices using manual contouring and the Scanco bone evaluation software. The threshold was set to 448 mg HA/cm^3^. In mice, 400 slices were used with a threshold of 180 mg HA/cm^3^.

### Flow Cytometric Analysis

Mesenteric lymph nodes and bone marrow from one tibia were collected from NTG and B27 rats. Cell suspensions were prepared and resuspended in RPMI 1640 media (Gibco) containing 10% fetal bovine serum, 2% sodium pyruvate, 0.05 mM 2‐mercaptoethanol, and 5 mM HEPES. To determine intracellular cytokine production, cells were stimulated with PMA and ionomycin (both at 500 ng/ml) in the presence of brefeldin A (10 μg/ml) for 4 hours. First, cells were stained with live-dead fixable reagent (Thermo Fisher) to exclude dead cells from the analysis. Second, surface markers were labeled using antibody mixtures prior to fixation and permeabilization with Foxp3/Transcription Factor Staining Buffer Kit according to the manufacturer instructions (Tonbo Biosciences). Surface marker antibodies were CD45 (OX-1 clone, BD-Biosciences), CD3 (1F4 clone, BD-Biosciences), TCRαβ (R73 clone, BD-Biosciences), and CD4 (Ox35 clone, Invitrogen). Then, cells were stained for intracellular IL-17 (TC11-18H10 clone, BD Horizon) and Foxp3 (FJK-16S clone, Invitrogen) for 30 minutes at 4°C. Eight-parameter cytometry was performed on an LSR III Fortessa flow cytometer (BD Biosciences) with FACSDiva software and analyzed with FlowJo software version 10.6.2.

### Adipocyte Cell Culture

3T3-L1 adipogenic cells were obtained from ATCC and maintained in DMEM supplemented with 10% FCS and 1% penicillin/streptomycin. Cells were deposited on glass coverslips at 70% confluence and differentiated into adipocytes using maintenance medium supplemented with 174 nM insulin, 0.5 mM 3-Isobutyl-1-methylxanthine (IBMX), 250 nM dexamethasone, and 2 µM rosiglitazone. Cells were kept in this medium for 3 days. Afterward, cells were switched to maintenance medium plus 174 nM insulin for another 3 days (post-differentiation). Thereafter, cells were kept in maintenance medium until day 10. Adipocytes were stained with LipidTox (Thermo Fisher Scientific) according to the manufacturer’s instructions.

To determine the effects of IL-17 on adipogenic differentiation, recombinant mouse IL-17A (R&D Systems) was added at the start of the differentiation protocol at various concentrations (10-100 ng/ml) for the entire differentiation period.

To determine the effect of IL-17 stemming from the serum of B27 transgenic rats, we added 5% of serum from NTG or B27 rats on day 3 of adipogenic differentiation for 4 days or on day 2 post-differentiation for 2 days. Additionally, some wells received 5 µg/ml of a neutralizing anti-mouse-IL-17A antibody or anti-IgG (both from R&D Systems). The percentage of rat serum was determined beforehand using a dose-response curve of NTG serum (0-5-25-50%) by assessing its effects on adipogenesis.

### Gene Expression Analysis

Long bones of NTG and B27 rats were flushed and bone marrow was harvested for total RNA isolation using TRIzol reagent (Invitrogen, Germany) following the manufacturer’s instructions. Total RNA from murine 3T3-L1 adipogenic cells was isolated with the High Pure RNA Isolation Kit (Roche, Germany) according to the manufacturer’s protocol and quantified using the NanoDrop spectrophotometer (Peqlab, Erlangen, Germany). Five hundred nanograms of RNA were reverse transcribed using M-MLV RT RNase (H-) Point Mutant (Promega, Mannheim, Germany) followed by SYBR Green-based quantitative real-time PCR according to established protocols (ABI7500 Fast; Applied Biosystems, Carlsbad, CA). The primer sequences were: *rActb* S: GCTACAGCTTCACCACCACA, *rActb* AS: AGGGCAACATAGCACAGCTT; *mActb* S: GATCTGGCACCACACCTTCT, *mActb* AS: GGGGTGTTGAAGGTCTCAAA; *rAdipoq* S: AGGAAACTTGTGCAGGTTGG, *rAdipoq* AS: CCTGTCATTCCAGCATCTCC; *mAdipoq* S: AAAGGAGAGCCTGGAGAAGC, *mAdipoq* AS: GTAGAGTCCCGGAATGTTGC; *rAdipor1* S: CTTCTACTGCTCCCCACAGC, *rAdipor1* S: ACACCACTCAAGCCAAGTCC; *mAp2* S: GATGCCTTTGTGGGAACCTG; *mAp2 AS:* GAATTCCACGCCCAGTTTGA; *mCebpa S:* CTGAGAGCTCCTTGGTCAAG, *mCebpa AS:* GAATCTCCTAGTCCTGGCTTG; *rPparg* S: CCGAGAAGGAGAAGCTGTTG, *rPparg* AS: TCAGCGGGAAGGACTTTATG; *mPparg* S: CACTCGCATTCCTTTGACATC, *mPparg A*S: CGCACTTTGGTATTCTTGGAG; (r = rat; m = mouse). PCR conditions were: 95°C for 2 min followed by 40 cycles with 95°C for 15 sec and 60°C for 1 min. Melting curves were assessed to confirm the amplification of one PCR product by increasing the temperature from 65°C to 95°C. Results were calculated based on the ΔΔCT method and are depicted in x-fold change normalized to the housekeeping gene *Actb*.

### Serum Analysis

Serum levels of glucose, triglycerides, cholesterol, and high- and low-density lipoprotein (HDL, LDL) were measured in B27 rats using a Roche ModularPPEanalyzer. Serum IL-17 and adiponectin levels in B27 rats were quantified using Quantikine ELISAs from R&D Systems.

### Adipokine Profiling

Rat serum was further used to quantify obesity-related cytokines using the Proteome Profiler Rat Adipokine Array Kit from R&D Systems according to the manufacturer’s protocol.

### Statistical Analysis

Results are presented as individual dots with the mean indicated as horizontal line. Comparisons were performed using unpaired Student’s *t*-test, two way ANOVA, and one‐way ANOVA followed by Tukey’s *post hoc* test as appropriate, by using GraphPad Prism version 7.0. P-values ≤ 0.05 were considered statistically significant.

## Results

### Loss of Bone Marrow Adipocytes in HLA-B27 Transgenic Rats and SKG Mice

**Figure 1:**
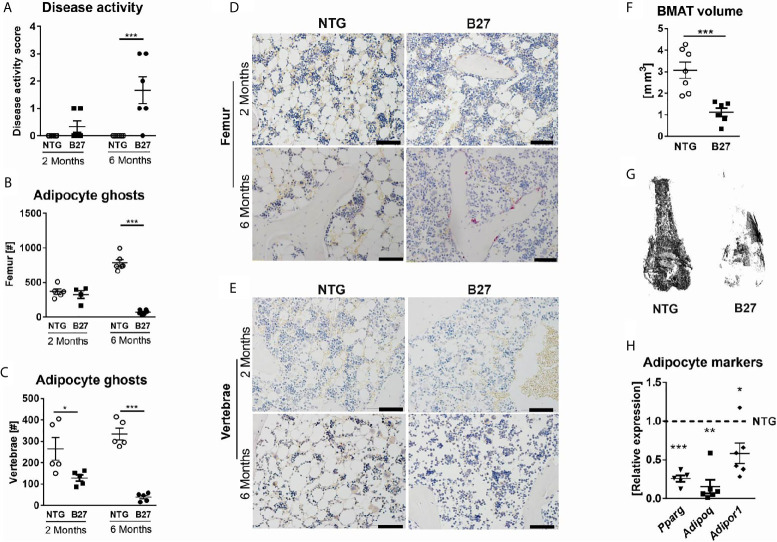
Decreased numbers of bone marrow adipocytes in B27 transgenic rats. **(A)** Clinical score of 2 and 6 months old NTG and B27 rats. Disease level was graded from 0-3: 0=no inflammation, 1=arthritis on one leg, 2=arthritis on both legs, 3=severe arthritis, and other organ manifestation. **(B)** Number of bone marrow adipocytes ghosts from the femur of 2- and 6 months old NTG and B27 (n=5-6 per group). **(C)** Number of bone marrow adipocytes ghosts from the vertebrae of 2- and 6 months old NTG and B27 (n=5 per group). **(D, E)** Representative histology sections from femur and vertebrae of 2-and 6 months old NTG and B27 rats stained with von Kossa/toluidine blue (scale bar: 100 µm) **(F, G)** Osmium tetroxide staining for bone marrow adipose tissue (BMAT) followed by micro-computed tomography (μCT) analysis. Quantification of fat volume **(F)** and representative images **(G)** of stained lipids in femora of NTG and B27 rats (n=6-7 per group). **(H)** Relative expression of PPAR*γ*, adiponectin, and adiponectin receptor in the bone marrow of NTG and B27 rats. Results are presented as individual dots with the mean indicated as horizontal line (n=5-6 per group). *p < 0.05; **p < 0.01 and ***p ≤ 0.001 *vs.* controls *via* unpaired Student’s t-test.

In the course of previous analyses that were aimed at investigating the rate of bone loss during disease progression in B27 rats (23), we observed that BMAds were strongly reduced along with disease development. To investigate this further, we examined the BMAT content in 2-and 6-month-old B27 rats with a low degree of inflammation and established disease (**Figure 1A**), respectively, using histology and osmium-based µCT, in which osmium stains lipid droplets and due to its heavy metal characteristics can be quantified using µCT. Histological analysis revealed no difference in the number of adipocytes in the femur of 2-month-old B27 rats, while 90% of BMAT reduction was observed in the 6-month-old cohort (**Figure 1B**). Analysis of the lumbar vertebral body also showed diminished BMAT in B27 rats before and after the onset of the disease (**Figure 1C**). Interestingly, loss of adipocytes was accompanied by an increased immune cell infiltration in the femur (6-month-old group) and lumbar vertebral body (2- and 6-month-old groups) of B27 rats (**Figures 1D, E**). µCT analysis revealed a more than a three-fold reduction in BMAT in the bone marrow of 6-month-old B27 rats compared to NTG rats (**Figures 1F, G**). Furthermore, the mRNA expression of adipocyte markers including peroxisome proliferator-activated receptor gamma (*Pparg*), adiponectin (*Adipoq*), and adiponectin receptor 1 (*Adipor1*) was strongly downregulated in the bone marrow of 6-month-old B27 rats (**Figure 1H**).

**Figure 2:**
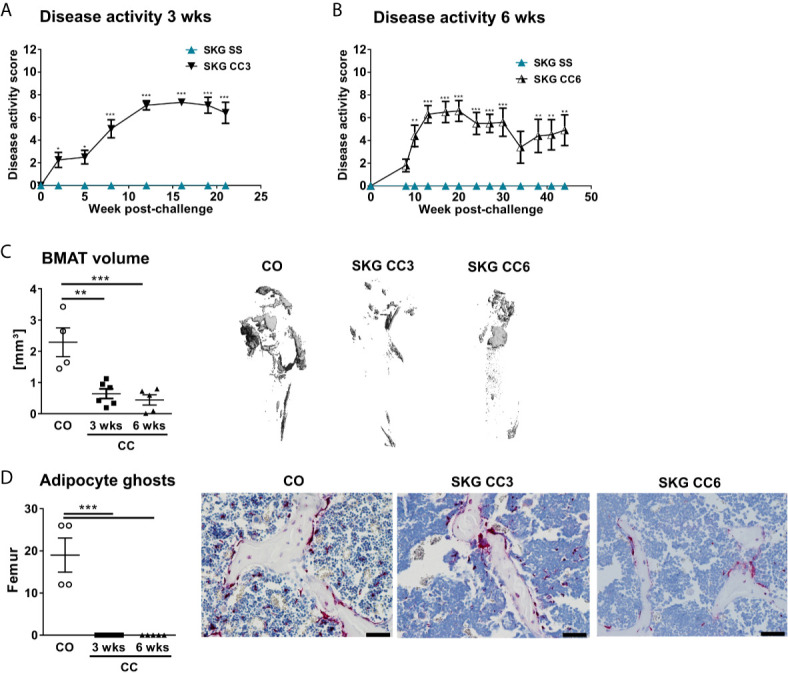
Decreased numbers of bone marrow adipocytes in SKG mice. **(A, B)** Clinical score of SKG animals at steady-state (SS) or curdlan-challenged (CC) for 3 and 6 weeks. **(C)** Osmium tetroxide staining for BMAT followed by μCT analysis. Quantification of fat volume (left) and representative images (right) of stained lipids in femora of SKG mice at 3- and 6-weeks post-curdlan challenge *vs.* control group. **(D)** Number of bone marrow adipocytes ghosts (left) and representative histology sections (right) from femur in SKG mice at 3 and 6 weeks post-curdlan challenge *vs.* control group stained with von Kossa/toluidine blue (scale bar: 100 µm). Results are presented as individual dots with the mean indicated as horizontal line (n=4-6 per group). *p < 0.05, **p ≤ 0.01, and ***p ≤ 0.001 *vs.* controls *via* two-way ANOVA with Tukey’s *post hoc* test **(A, B)** and one-way ANOVA with Tukey’s *post hoc* test **(C, D)**.

To investigate if a similar phenotype occurs in another murine model of SpA, we assessed the BMAT in curdlan-challenged SKG mice. SKG mice developed severe inflammatory arthritis (**Figures 2A, B**) that was associated with a marked reduction in BMAT content (**Figure 2C**) and BMAd number (**Figure 2D**) at three weeks post curdlan challenge that remained low until six weeks. Thus, BMAT is severely reduced in two characteristic murine models of chronic inflammatory SpA.

### Chronic Inflammation in Murine Models of SpA Is Associated With Loss of Adipose Tissue and an Altered Metabolic Profile

**Figure 3:**
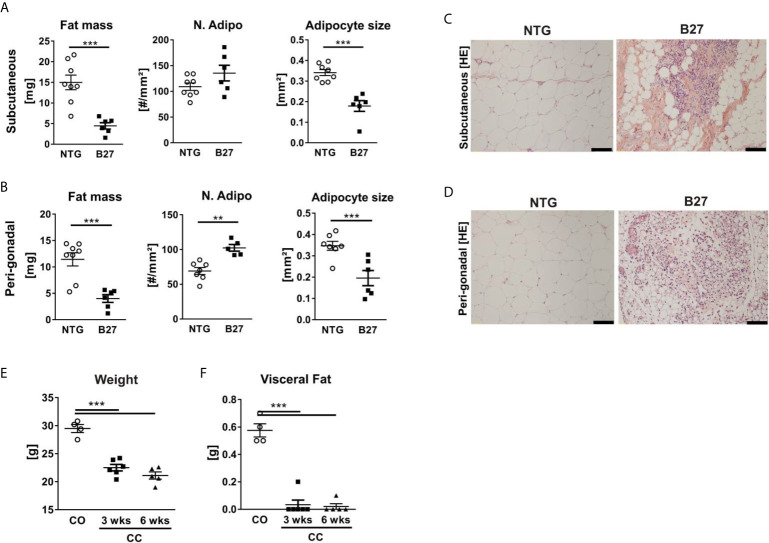
Chronic inflammation in rodent models of SpA leads to loss of white adipose tissue. **(A, B)** Fat mass, number, and size of adipocytes in subcutaneous (top) and peri-gonadal (bottom) adipose tissue of NTG and B27 rats. **(C, D)** Representative histology sections stained with hematoxylin/eosin staining. Scale bars: 100 μm (n=6-8 per group). **(E, F)** Bodyweight and visceral fat weight of SKG mice at 3- and 6-weeks post-curdlan challenge vs. control group (n=4-6 per group). Results are presented as individual dots with the mean indicated as horizontal line. **p ≤ 0.01, and ***p ≤ 0.001 *vs* controls *via* unpaired Student´s t-test **(A, B)** and one-way ANOVA with Tukey’s *post hoc* test **(E, F)**.

**Figure 4:**
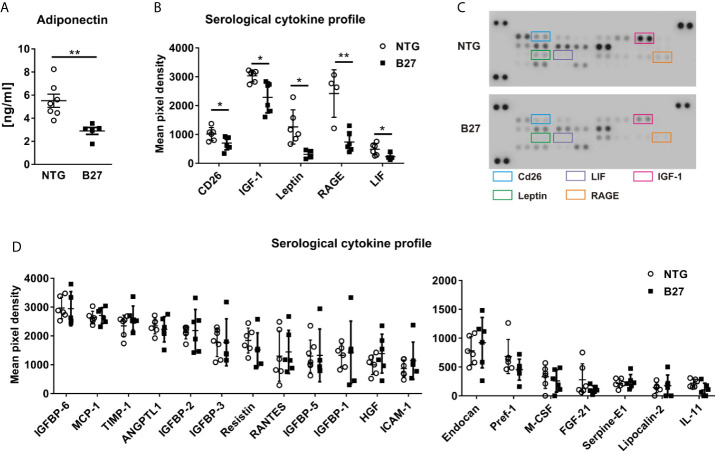
Altered metabolic profile in B27 transgenic rats. **(A)** Serum concentrations of adiponectin in NTG and B27 rats (n=5-7 per group). **(B)** Significantly regulated cytokines in the serum of NTG and B27 rats. **(C)** Representative image of cytokine profiler array results from serum of NTG and B27 rats. Significant ones are highlighted. **(D)** Relative levels of not-regulated obesity-related cytokines in NTG and B27 rats (B,D n=6-8 per group). Data are presented as mean ± SD. *p ≤ 0.05 and **p ≤ 0.001 NTG *vs.* B27 *via* unpaired Student’s t-test.

To further investigate whether the decreased amount of adipocytes in the bone marrow from B27 rats is site-specific, we measured in these rats body weight and subcutaneous and peri-gonadal fat weight. We observed a significant loss of body weight (**Table 1**) as well as a marked reduction of subcutaneous and peri-gonadal fat mass compared to NTG rats (**Figures 3A, B**). Histological analysis of these fat pads showed that adipocyte number was similar, but that adipocyte size was significantly reduced in B27 rats compared to NTG rats (**Figures 3A, B**). Furthermore, HE staining showed massive inflammatory infiltrates into the adipose tissue at both sites in B27 rats (**Figures 3C, D**). Importantly, these results were corroborated in arthritic SKG mice, showing weight loss (**Figure 3E**) and reduced subcutaneous fat mass (**Figure 3F**). Thus, loss of BMAT is not bone marrow-specific, but also white adipose tissue undergoes destruction during chronic inflammation, underscoring the consumptive or catabolic nature of unopposed chronic inflammation. In addition to adipose tissue loss, B27 rats showed significant reduction in serum triglycerides, cholesterol, LDL as well as HDL (**Table 1**). Moreover, blood glucose levels were decreased by 20% (**Table 1**). To further examine the levels of adipokines, we performed a proteome profiler assay on NTG and B27 rat sera. This analysis showed that adiponectin and leptin levels, two cytokines produced mainly by adipocytes, were highly reduced in B27 rats (**Figures 4A, B**). In addition, CD26, IGF-1, LIF, and RAGE were also significantly reduced (**Figure 4B**). Representative blots for cytokine expression in NTG and B27 are shown in **Figure 4C**. Of note, the abundance of all other obesity-related cytokines investigated in the serum was not altered (**Figure 4D**).

**Table 1:** Body weight and serum fat parameters in no-transgenic and HLA-B27 transgenic rats.

Parameters	NTG N = 8	B27 N = 6	p-value
Weight (g)	349 ± 101	248 ± 50.0	0.046
Serum glucose (mmol/L)	14.7 ± 2.25	11.8 ± 1.67	0.031
Triglycerides (mmol/L)	1.93 ± 0.87	1.00 ± 0.46	0.036
Cholesterol (mmol/L)	2.99 ± 0.98	1.65 ± 0.29	0.007
LDL (mmol/L)	0.37 ± 0.09	0.23 ± 0.11	0.032
HDL (mmol/L)	1.75 ± 0.36	1.04 ± 0.25	0.002

NTG, non-transgenic rat; B27, HLA-B27 transgenic rat; LDL, low-density lipoprotein; HDL, high-density lipoprotein. Data are represented as mean ± SD.

### Hematopoietic Composition of the Bone Marrow and Blood of B27 Rats

As dysregulated hematopoiesis occurs in several chronic inflammatory diseases, we analyzed immune cell populations present in the bone marrow and assessed hematological parameters in the blood. The frequency of neutrophils was significantly increased in the bone marrow, mostly due to an increase in the promyeloid stage and the segmented neutrophils (**Supplementary Figure 1A**). In contrast, lymphocytes and monocytes frequencies were decreased (**Supplementary Figure 1B**). Leukocyte numbers were increased by 3-fold in the peripheral blood of B27 animals, showing significantly increased numbers in neutrophils, eosinophils, monocytes, and platelets (**Table 2**). As expected, erythroid precursors in the bone marrow were significantly reduced, as was the hematocrit and the mean corpuscular volume, indicating that anemia of inflammation had developed in B27 rats (**Figure 1** and **Table 2**). Thus, during inflammation, neutrophils appear to account for most of the inflammatory cells in the bone marrow of B27 rats.

**Table 2:** Blood differential in no-transgenic and HLA-B27 transgenic rats.

Parameters	NTG N = 8	B27 N = 5	p-value
**Hematocrit (%)**	**47.6 ± 4.37**	**41.4 ± 3.50**	**0.025**
Hemoglobin (g/dl)	14.8 ± 2.68	14.2 ± 2.87	0.787
**MCV (fl)**	**53.4 ± 1.98**	**50.4 ± 2.07**	**0.029**
MCH (pg)	16.6 ± 2.00	16.4 ± 0.55	0.813
MCHC (g/dl)	31.4 ± 3.82	32.4 ± 1.34	0.567
Erythrocytes (10^5^ #/mm³)	88.9 ± 6.99	81.3 ± 9.08	0.117
**Leucocytes (10³ #/mm³)**	**4.22 ± 2.50**	**11.6 ± 1.35**	**<0.001**
Neutrophils (10³ #/mm³)	1.50 ± 1.30	20.6 ± 15.4	0.053
** Eosinophils (#/mm³)**	**26.5 ± 27.1**	**115 ± 13.7**	**<0.001**
Basophils (#/mm³)	22.5 ± 48.5	24.6 ± 55.0	0.944
Lymphocytes (10³ #/mm³)	2.36 ± 1.26	3.52 ± 0.96	0.109
** Monocytes (#/mm³)**	**59.3 ± 56.3**	**425 ± 241**	**0.002**
** Platelets (10^5^ #/mm³)**	**7.10 ± 1.31**	**9.60 ± 1.06**	**0.006**

NTG, non-transgenic rat; B27, HLA-B27 transgenic rat; MCV, mean corpuscular volume; MCH, mean corpuscular hemoglobin; MCHC,: mean corpuscular hemoglobin content. Data are represented as mean ± SD.

Bold values mean they are statistically significant.

### Elevated Levels of IL-17 May Contribute to the Loss of Adipocytes in B27 Rats

**Figure 5:**
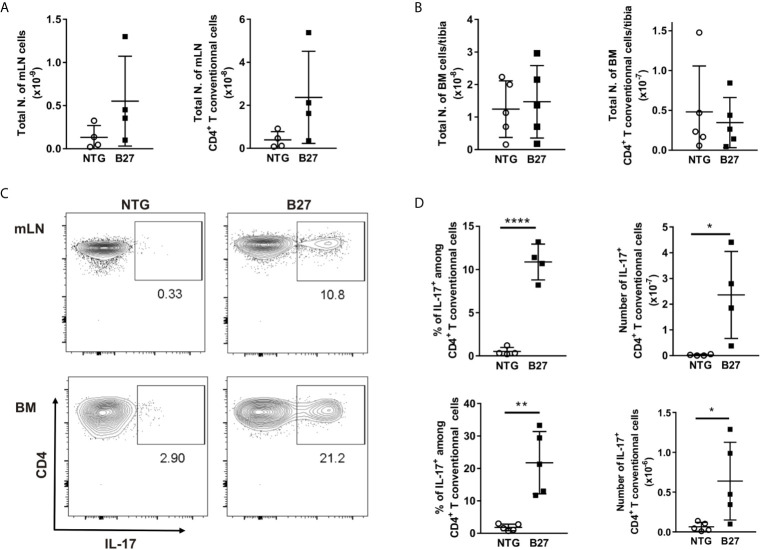
Expansion of IL17+ producing CD4+ T cells in the bone marrow from B27 transgenic rats. **(A)** Absolute number of live cells and CD4^+^ T cells in mesenteric lymph nodes (mLN) from NTG (grey) and B27 (white) rats were determined using flow cytometry. **(B)** Absolute number of live cells and CD4^+^ T cells in bone marrow (BM) from NTG and B27 rats. **(C)** Representative plots of intracellular IL-17 staining gated on CD4^+^ Foxp3^-^ T cells in mLN (top) and BM (bottom) from NTG (left panel) or B27 (right panel) rats. **(D)** Frequencies of IL-17^+^ cells among CD4^+^ T cells (left) and their absolute numbers (right) in mLN (top) and BM (bottom) from NTG and B27 rats (A-D n=4-5 per group). Results are presented as individual dots with the mean indicated as horizontal line. Data were analyzed by unpaired Student’s t-test (*p<0.05, **p < 0.01 and ****p < 0.0001).

**Figure 6:**
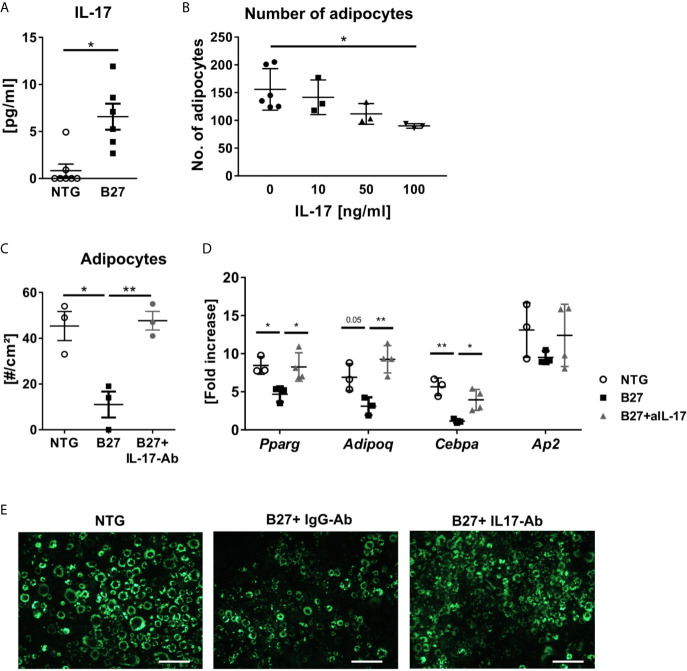
IL-17 from B27 transgenic rat serum inhibits adipogenesis in vitro. **(A)** IL-17 levels in sera of in B27 and NTG rats (n=6-7 per group). **(B)** Absolute numbers of 3T3 adipocytes after exposure to control medium or increasing doses of IL-17 during adipocyte differentiation (n=3-6 per group). **(C, D)** Absolute numbers of 3T3 adipocytes and relative expression of the most representative adipocyte markers after 48 h exposure to 5% serum from NTG rats, B27 rats + IgG antibody (IgG-Ab), or B27 rats + neutralizing anti-IL-17 antibody (IL-17-Ab) treatment (n=3-6 per group). **(E)** Representative fluorescence microscopy pictures of LipidTOX stained 3T3 cells. Scale bar: 50 µm. All experiments were performed independently three times. Results are presented as individual dots with the mean indicated as horizontal line. *p ≤ 0.05 and **p ≤ 0.01 NTG *vs.* B27 *via* unpaired Student’s t-test **(A)**. and one-way ANOVA with Tukey’s *post hoc* test **(B–D)**.

As B27 rats have been shown to have an increased prevalence of CD4^+^ T-helper 17 (Th17) cells and elevated serum levels of IL-17 (24, 25), we investigated the absolute number of CD4^+^ T present in the bone marrow and used mesenteric lymph nodes (mLN) as a control with a known expansion of Th17 cells. Absolute numbers of CD4^+^ T cells were significantly higher in mLN of spondyloarthritic B27 compared to the healthy NTG rats (**Figure 5A**), while similar numbers were found in the bone marrow (**Figure 5B**). Importantly, the frequency of IL-17-producing CD4^+^ T cells was significantly increased in both mLN and bone marrow of B27 rats compared to NTG rats (**Figures 5C, D**). Accordingly, B27 rats showed an 8-fold higher serum level of IL-17 than NTG (**Figure 6A**). Given that IL-17 reduces preadipocyte differentiation and alters adipogenesis (26, 27), we examined whether this cytokine could be involved in the depletion of BMAT in our model. For this, we first tested whether IL-17 indeed suppresses adipogenesis of 3T3 adipocytes. Treatment of 3T3 cells with IL-17 during the differentiation period reduced in a dose-dependent manner adipocyte numbers (**Figure 6B**). Importantly, treatment of 3T3 cells with 5% serum from B27 rats starting at day 3 of differentiation reduced adipocyte number and the mRNA expression of adipocyte markers compared to cells treated with 5% NTG serum (**Figures 6C, D**). This effect with sera was reversed when cells were incubated with a neutralizing IL-17 antibody confirming the critical role of IL-17 in the regulation of adipocyte differentiation (**Figure 6E**). Notably, treating adipocyte cultures on day 2 post-differentiation with rat serum did not result in altered adipocyte numbers (NTG: 120.7 ± 13.7 *vs.* B27: 101.3 ± 38.9 adipocytes, p=0.43). Thus, these data suggest that the high amount of IL-17 present in the serum of B27 reduces adipogenesis.

## Discussion

The ability of BMAds to concurrently influence bone remodeling and hematopoiesis (7, 8) places them in a crucial position in the regulation of bone and immune cell fate. Notably, recent evidence demonstrated that BMAds play a decisive role in regulating immune responses, contributing to increased levels of pro-inflammatory cytokines and oxidative stress (6). However, how chronic inflammation affects BMAT is currently unknown. We previously observed a drastic reduction of BMAd with disease duration in B27 rat, while studying the impact of chronic inflammation on bone in this model of SpA (23). Here, we examined in more detail the femoral and vertebral BMAT content in 2 and 6 months old B27 rats. Using 3D µCT and histology, we showed that BMAT was reduced by up to 90% in 6-month-old B27 rats with established disease in the femur and vertebral body compared to control NTG. This finding was not restricted to the B27 rat model of SpA, since similar findings were also observed in curdlan-challenged SKG mice, suggesting that depletion of BMAT is a common characteristic of SpA rodent models.

Weight loss and decrease in lean mass have been commonly described in individuals with inflammatory rheumatic disorders, reflecting their consumptive nature (28, 29). Based on this clinical evidence, we quantified total body weight as well as subcutaneous and peri-gonadal fat weight to assess whether the loss of BMAds is site-specific. Both B27 rats and SKG mice showed a significant body weight and overall fat mass loss. Thus, these data show that the decreased amount of BMAT is not a localized, but rather a systemic phenomenon, and that also white adipose tissue decreases during chronic inflammation. Consistently, proteomic analyses showed low serum levels of adipokines in B27 rats compared to NTG. Interestingly, reduction of both adiponectin and leptin has been associated with increased inflammatory responses and enhanced susceptibility to the toxicity of proinflammatory stimuli, respectively (29, 30).

In addition, high inflammation reduces circulating levels of lipids (31–33). Analysis of the serum lipid profile in B27 rats revealed marked dyslipidemia with decreased triglycerides, cholesterol, and associated lipoproteins as compared to the controls. Reduction in HDL cholesterol levels in our rat model appeared consistent with clinical observations previously reported (34). Indeed, patients affected by axial SpA and psoriatic arthritis present with an abnormal lipid profile and low HDL levels, which have been linked to accelerated manifestations of cardiovascular diseases and reduced lifespan (35). Nevertheless, the molecular factors that potentially mediate the interaction between inflammatory disease and altered lipid metabolism require further investigations. A plausible explanation is that proinflammatory cytokines may redirect energy consumption in favor of hematopoietic cells at the expense of fat tissue, thereby guaranteeing that enough energy can be provided to immune cells in states of high demand, such as during inflammation (36). Thus, cytokines might have direct effects on lipid metabolism as well as glucose metabolism, which can also explain the significant reduction of blood glucose levels in B27 rats as compared to the healthy controls.

Although the immune cell network that drives SpA is still unclear, experimental evidence supports a central role for HLA-B27 expressing hematopoietic cells in disease development (37). Therefore, to gain a greater understanding of the hematopoietic cell composition and explore which other cells inhabit the bone marrow instead of the BMAds, we analyzed immune cell populations in B27 rats. Systemic inflammation was associated with an increased number of mature neutrophils, which was mirrored by decreased erythroid precursors. These data support previous findings, in which non-resolving joint and intestinal inflammation in the SKG mouse model of SpA was accompanied by dysregulated hematopoietic stem cell activity and biased differentiation toward myeloid progenitors, culminating in an invasion of target organs by activated neutrophils (38). Within the same study, the authors demonstrated that CD4^+^ T-cell depletion was not required for the development of SpA in this model, supporting the critical function of innate versus adaptive immune response in inflammation (38). Nevertheless, the critical contribution of adaptive cells to the disease has been shown in other situations such as in HLA-B27 transgenic rats (12). This leads to the production of proinflammatory cytokines implicated in the pathogenesis of SpA. In particular, IL-17 derived from CD4^+^Th17 has been found to positively correlate with disease severity. Indeed, serum concentrations of IL-17 were higher in patients with SpA than in age and sex-controlled healthy individuals (39, 40). These observations are congruent with our analysis, where B27 rats displayed higher levels of IL-17 in the serum as compared to the NTG controls. In addition, insights into the molecular regulation of bone homeostasis revealed that IL-17 steers mesenchymal stem cells into an osteogenic fate and promote osteoclastogenesis in a RANKL-dependent manner (16, 41). Further, *in vivo* studies implicate IL-17 in the regulation of adipogenesis by inhibiting preadipocyte differentiation, hampering metabolic function of mature adipocytes, and consequentially reducing white adipose tissue accumulation (16, 42). Accordingly, our *in vitro* experiment confirmed a significant reduction in the number of adipocytes after treatment with recombinant IL-17 as well as after treatment with B27 rat serum. This effect was only observed, however, when starting the treatment early during the differentiation process. The reduction of adipocytes was dependent on the IL-17 present in the serum of B27 rats, as suppression of adipogenesis was reversed when IL-17 was neutralized using antibodies. Whether IL-17 mediates the reduction of BMAT during chronic inflammation in SpA *in vivo* and whether this is driven by suppression of BMAT expansion and/or loss of mature BMAds remains to be investigated.

Besides its prominent role in the regulation of adipose tissue and energy homeostasis, IL-17 has been shown to activate innate immune mechanisms, including the recruitment and survival of neutrophils (13, 43, 44). Thus, the high levels of IL-17 could explain the reduction of BMAds and the increase of neutrophils within the bone marrow in our rat model. In fact, the abundance of IL-17-producing CD4^+^ T cells observed in the bone marrow of B27 rats may promote the accumulation of neutrophils and, simultaneously, induce lipolysis in the surrounded adipocytes to provide energy for the immune system. Clearly, further investigations are necessary to thoroughly address this hypothesis and clarify the mechanisms and purpose of BMAd reduction during chronic inflammation.

Despite strong evidence that BMAT is reduced in rodent models of SpA, our study has potential limitations. First, our animal models do not fully reflect the spectrum of spine manifestations found in patients with SpA. For example, osteophytes and fatty streaks at the vertebral column are rarely observed. Moreover, we evaluated adipokines in the serum. However, other fat depots such as white adipose tissue also produce adipokines that are released into the circulation. Therefore, it would be interesting to analyze adipokine concentrations directly in the bone marrow microenvironment to better address which adipokines are locally altered by chronic inflammation.

## Conclusions

Our results show a significant reduction of white adipose tissue and BMAT in two relevant rodent models of SpA. Elevated serum levels of IL-17 suggest a potential role of IL17^+^CD4^+^ T cells in sustaining inflammation by disrupting BMAT.

## Data Availability Statement

The raw data supporting the conclusions of this article will be made available by the authors, without undue reservation.

## Ethics Statement

The animal study was reviewed and approved by APAFIS-8910 and UK Home Office project license (PBFB4BA22).

## Author Contributions

MR, LA, MBr, and LH designed the experiments. GF, MR, UB, IF, MBe, SG, MvB, and LA performed all experiments and analyzed the data. MBr, SG, and MvB evaluated bone marrow. NH performed SKG mouse experiments. All authors contributed to data discussion and interpretation. GF and MR drafted the manuscript. All authors contributed to the article and approved the submitted version.

## Funding

This work was supported by grants from the Deutsche Forschungsgemeinschaft and the European Union’s Horizon 2020 research and innovation programme under the Marie Skłodowska-Curie grant agreement No 860898 to MR and LH, as well as NH, supported by Versus Arthritis Grant No. 20372.

## Conflict of Interest

The authors declare that the research was conducted in the absence of any commercial or financial relationships that could be construed as a potential conflict of interest.

## Supplementary Material

Supplementary Figure 1 Effect of chronic inflammation on blood cells in the bone marrow of B27 rats. Percentage of neutrophil subpopulations **(A)**, other leucocyte populations **(B)**, and erythroblast populations **(C)** in the bone marrow of NTG and B27 rats (n=6-8 per group). Data are presented as mean ± SD. *p ≤ 0.05, **p ≤ 0.01, and ***p ≤ 0.001 NTG *vs.* B27 *via* unpaired Student´s t-test.
