# Supplementary figures and images for: Rodent Models of Spondyloarthritis Have Decreased White and Bone Marrow Adipose Tissue Depots
Source: Front Immunol. 2021 Jun 2;12:665208. doi: 10.3389/fimmu.2021.665208 (PMC8207134; doi:10.3389/fimmu.2021.665208)

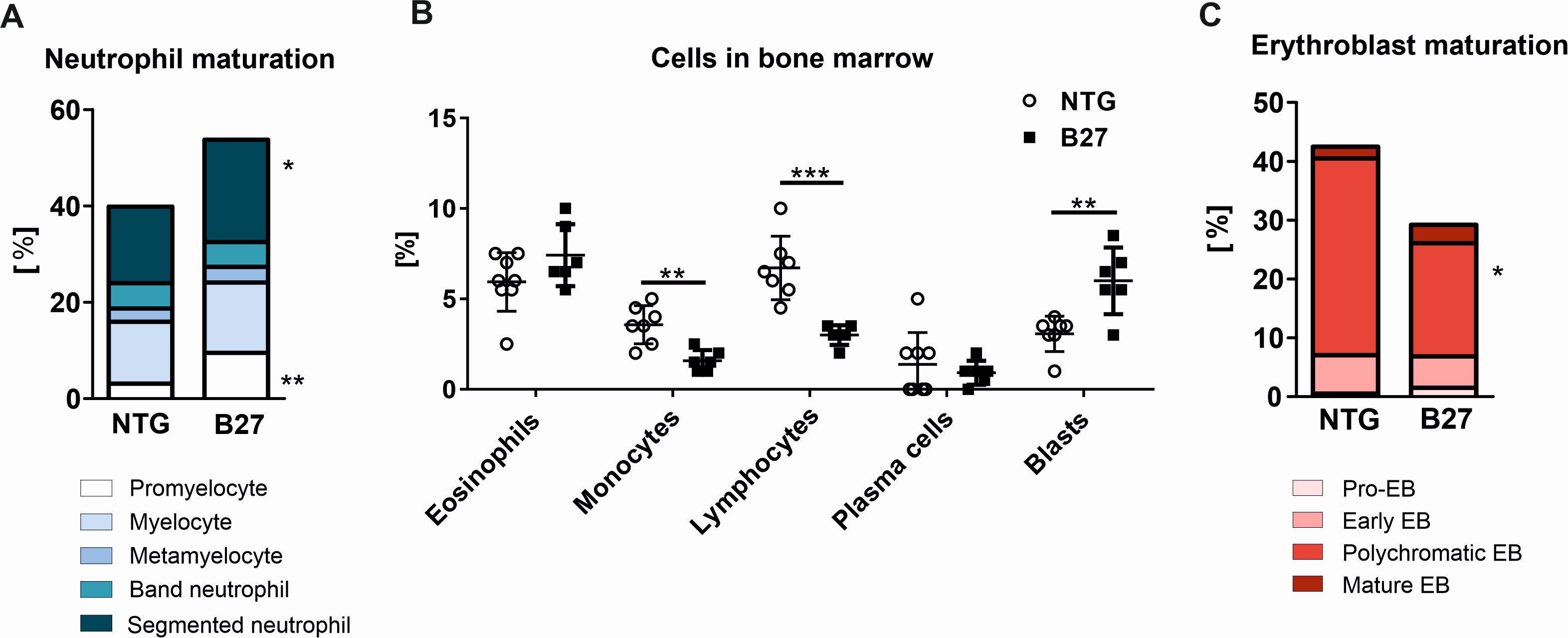

Supplement: Supplementary Figure 1 — Effect of chronic inflammation on blood cells in the bone marrow of B27 rats. Percentage of neutrophil subpopulations (A), other leucocyte populations (B), and erythroblast populations (C) in the bone marrow of NTG and B27 rats (n=6-8 per group). Data are presented as mean ± SD. *p ≤ 0.05, **p ≤ 0.01, and ***p ≤ 0.001 NTG vs. B27 via unpaired Student´s t-test. [file Image_1.jpg]
